# NSAIDs attenuate hyperalgesia induced by TRP channel activation

**DOI:** 10.1016/j.dib.2015.12.055

**Published:** 2016-01-13

**Authors:** Ivliane Nozadze, Nana Tsiklauri, Gulnaz Gurtskaia, Merab G. Tsagareli

**Affiliations:** Laboratory of Pain and Analgesia, Beritashvili Center for Experimental Biomedicine, Tbilisi, Georgia

**Keywords:** Allodynia, Cold pain, Heat pain, Hyperalgesia, Signal transduction, Nociception

## Abstract

Transient receptor potential (TRP) cation channels have been extensively investigated as targets for analgesic drug discovery. Because some non-steroidal anti-inflammatory drugs (NSAIDs) are structural analogs of prostaglandins (mediators of inflammation) and NSAIDs attenuate heat nociception and mechanical allodynia in models of inflammatory and neuropathic pain, we examined three widely used NSAIDs (diclofenac, ketorolac, and xefocam) on the activation of TRPA1 and TRPV1 channels using thermal paw withdrawal (Hargreaves) test and mechanical paw withdrawal (von Frey) test in male rats. Thermal withdrawal latencies and mechanical thresholds for both hind paws were obtained with 5, 15, 30, 45, 60, and 120 min intraplantar post-injection of TRPA1 agonizts, allyl isothiocyanate (AITC) (a natural compound of mustard oil) and cinnamaldehyde (CA), and TRPV1 agonist capsaicin or vehicle. Twenty minutes prior to the start of the experiment with TRP agonizts, diclofenac, ketorolac or xefocam were pre-injected in the same hindpaw and animals were examined by these two tests. After pretreatment of all three NSAIDs in the ipsilateral (injected) hindpaw that produced strong antinociceptive effects, AITC, CA, and capsaicin caused significant decreases in latency of the thermal withdrawal reflex compared with vehicle or the contralateral hindpaw. The same findings were observed for the paw withdrawal threshold. In approximately 30 min the effects of CA, AITC, and capsaicin returned to baseline. The data are different from our previous evidence, where TRPA1 agonizts AITC and CA and TRPV1 agonist capsaicin produced hyperalgesia for nearly 2 h and resulted in facilitation of these withdrawal reflexes (Tsagareli et al., 2010, 2013). Thus, our data showing that NSAIDs suppress thermal and mechanical hyperalgesia following TRP activation could presumably due to inactivation or desensitization of TRPA1 and TRPV1 channels by NSAIDs.

**Specifications table** *[please fill in right-hand column of the table below]*

**Table:** 

Subject area	*Life Sciences*
More specific subject area	*Neuropharmacology*
Type of data	*text file, figures*
How data was acquired	*Behavioral study instruments*
Data format	*Raw*
Experimental factors	*Latency of thermal paw withdrawal latency and mechanical paw withdrawal threshold.*
Experimental features	*Some commonly used NSAIDs, such as clodifen, ketorolac, and xefocam attenuate TRPA1 channel activation to enable treatment with cinnamaldehyde (CA) and allyl isothiocyanate (AITC), and TRPV1 channel activation to enable treatment with capsaicin.*
Data source location	*Tbilisi, Georgia Republic*
Data accessibility	*The data are supplied with this article*

**Value of the data**

• We examined three widely used non-steroidal anti-inflammatory drugs (NSAIDs) (diclofenac, ketorolac, and xefocam) on the activation of transient receptor potential (TRP) channels (TRPA1 and TRPV1) to its agonizts, allyl isothiocyanate (AITC, main compound of mustard oil), cinnamaldehyde (CA), and capsaicin in behavioral experiments in rats.
• We found that ketorolac pretreatment inactivates TRPA1 channels to its agonizts, AITC and CA, and results in the return of hyperalgesia to baseline in approximately 30 minutes.
• In the same manner pretreatment with all three NSAIDs inactivates TRPV1 channel to its agonist capsaicin.

• Our findings show for the first time an attenuation of TRPA1 and TRPV1 activation by NSAIDs to channel agonizts, cinnamaldehyde, mustard oil, and capsaicin in two behavioral assays.

• TRP cation channels could be investigated as promising targets for analgesic drug discovery.

## 1 Data

Transient receptor potential (TRP) cation channels serve as cellular sensors for a wide spectrum of physical and chemical stimuli, such as temperature, cyclic nucleotides, phosphorylation potential, osmotic pressure, and some beneficial and harmful environmental inputs [1], [2], [5], [7], [11], [12].

Recent data in the field of pain research have established a subset of thermo-TRP channels that are capable of initiating sensory nerve impulses following the detection of thermal, as well as mechanical and chemical irritant stimuli. These irritants include menthol, capsaicin, cinnamaldehyde (CA), mustard oil, and others [3], [4], [6], [8], [10].

**Fig. 1:**
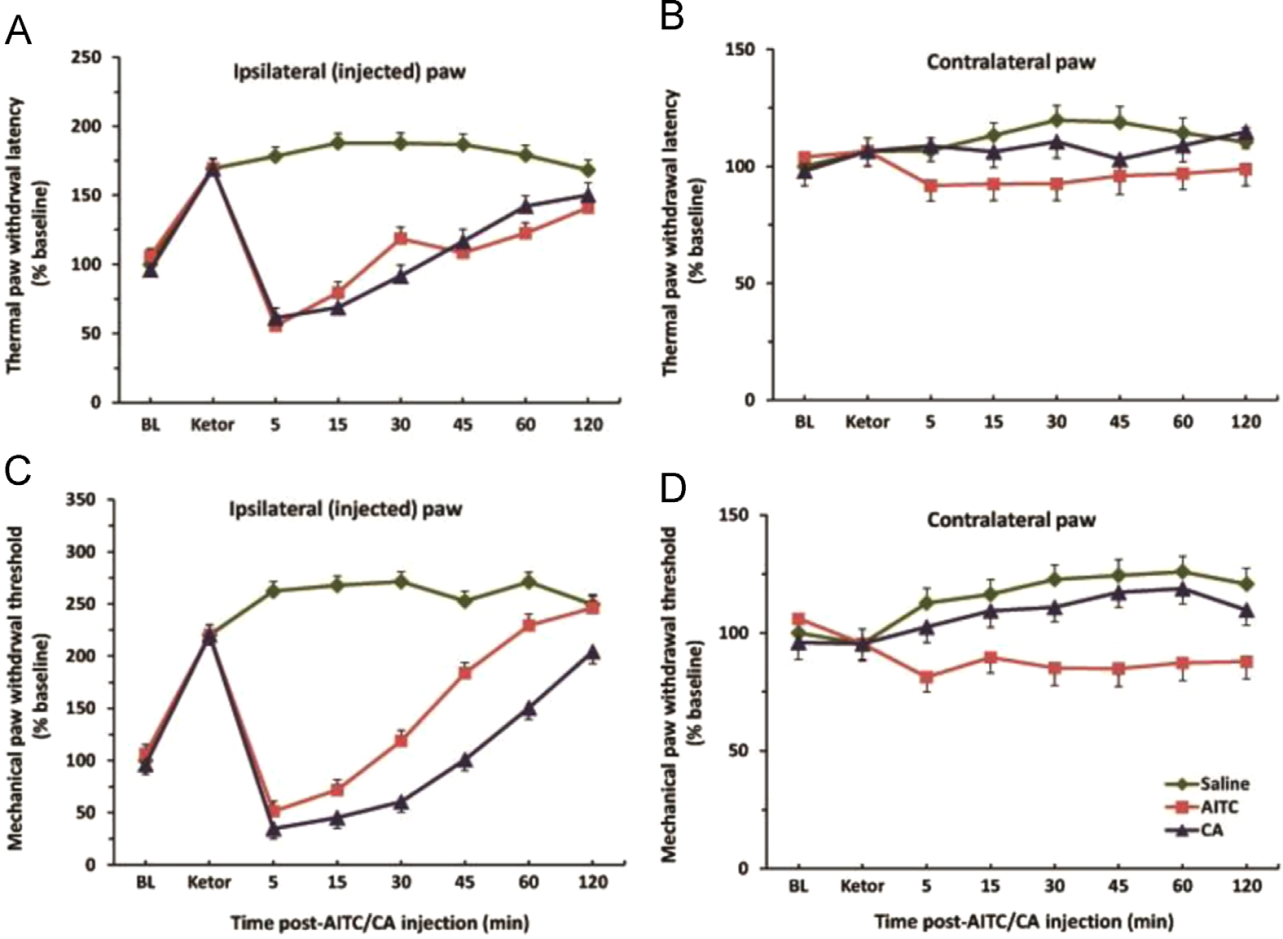
(A) Dynamics of the thermal paw withdrawal latency after ketorolac pretreatment following ipsilateral intraplantar injection of vehicle, AITC and CA. There were significant effects in AITC and CA groups *vs.* vehicle control and contralateral (non-injected) paw (B) for the first 30 min (P).

One of the current mainstays of pain therapy is agents like non-steroidal anti-inflammatory drugs (NSAIDs) which are specified with some side effects [9]. Here we report, using behavioral tests in rats that one commonly used NSAIDs such as ketorolac, inactivate TRPA1 channel to enable treatment with CA and allyl isothiocyanate (AITC), a natural compound of mustard oil (Fig. 1). In particular, pretreatment with ketorolac resulted in a strong antinociceptive effect (P<0.001). Subsequent injections with AITC and CA caused significant decreases in the latency of the thermal withdrawal reflex in the ipsilateral (injected) hindpaw for about first 30–40 min (Fig. 1A). The same findings we observed for the paw withdrawal threshold with mechanical stimulation (Fig. 1C). In approximately 40 min, the effects of CA and AITC returned to baseline. These data are different from our previous results, where TRPA1 channel agonists CA and AITC produced hyperalgesia for nearly 2 h and facilitated thermal and mechanical withdrawal reflexes [8], [10].

**Fig. 2:**
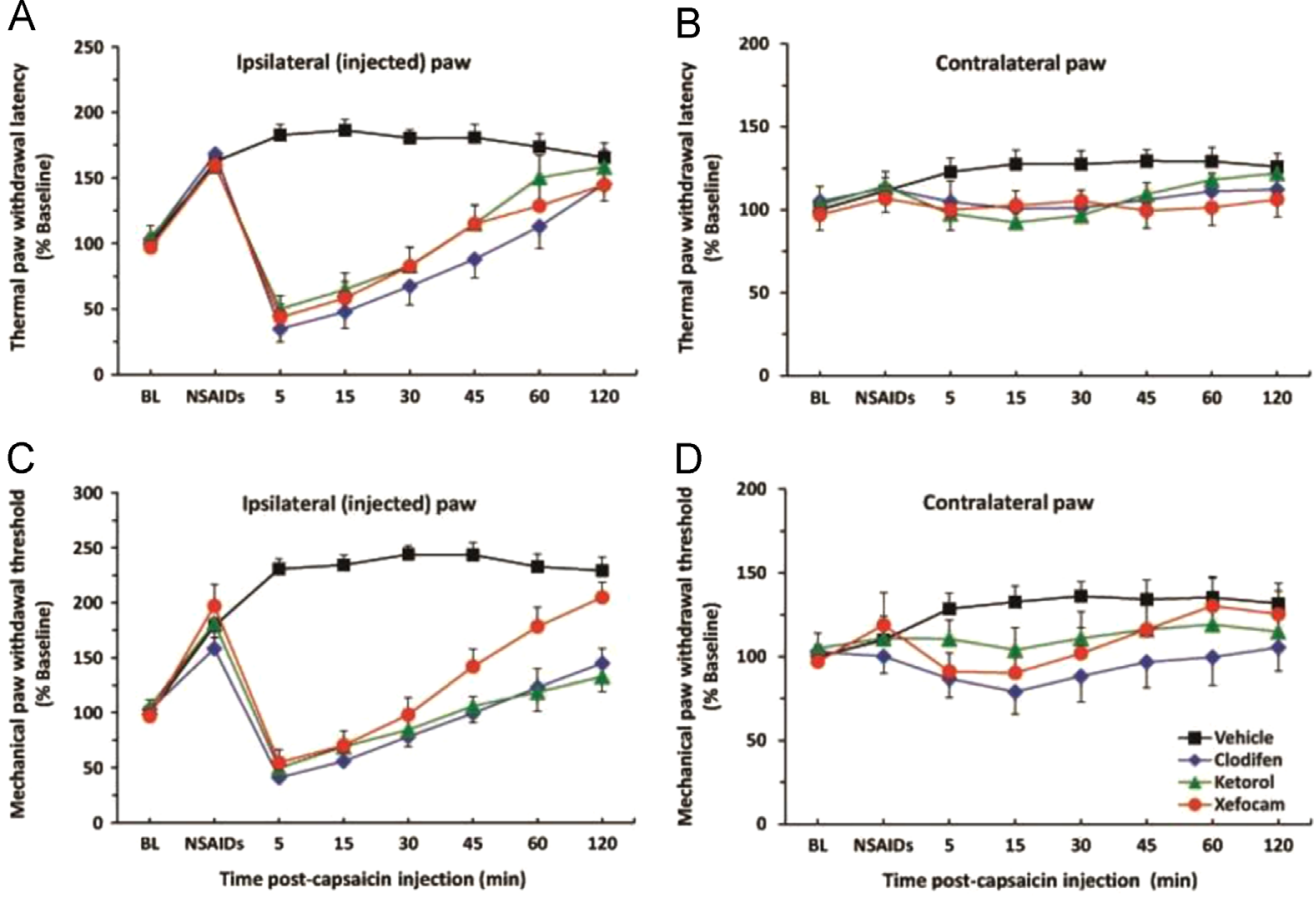
(A) Dynamics of the thermal paw withdrawal latency after NSAIDs (clodifen, ketorolac and xefocam) pretreatment following ipsilateral intraplantar injection of vehicle and capsaicin. There were significant effects in capsaicin groups *vs.* vehicle control and contralateral (non-injected) paw (B) for the first 30 min (P).

Concerning the TRPV1 channel we have found that three commonly used NSAIDs, such as diclofenac, ketorolac, and xefocam attenuate hyperalgesia induced by TRPV1 agonist capsaicin after 40–45 min of its injection (Fig. 2A). Equivalent findings were observed for the paw withdrawal threshold to mechanical stimulation (Fig. 2C). In both tests, the effects of capsaicin returned to baseline in approximately 45 min.

We suggest that presented data indicate a novel mechanism involving the anti-inflammatory and analgesic effects of NSAIDs, which may be involved in direct inactivation or desensitization of TRPA1 and TRPV1 channels and could be used for development novel class of analgesics.

## 2 Experimental design, materials and methods

### 2.1 Experimental design

Behavioral measurements were performed using thermal paw withdrawal (Hargreaves) test and mechanical paw withdrawal (von Frey) test in rodents after pretreatment their hindpaws with NSAIDs and subsequent treatment with TRP channel agonists.

### 2.2 Animals

Experiments were carried out using adult male Wistar rats (350–450 g), which were singly housed and given rodent chow and water *ad libitum*. The Beritashvili Experimental BMC Animal Care and Use Committee approved the study protocol. Every effort was made to minimize both the number of animals used and their suffering. Guidelines of the International Association for the Study of Pain regarding animal experimentation were followed throughout [13].

### 2.3 Application of chemicals

AITC (15%), CA (20%), and capsaicin (0.4%) (Sigma-Aldrich, St Louis, MO, USA) or vehicle control (mineral oil, or Tween 80, Fisher Scientific, USA) were injected intraplantar with a 30 gauge needle. For thermal and mechanical paw withdrawal tests, AITC, CA, capsaicin, or vehicle (2 μl) was applied to one hind paw. Twenty minutes prior to apply these TRP agonists, the same volume of NSAIDs, diclofenac (2.5%), ketorolac (3%), and xefocam (0.4%) were injected in the same hindpaw and animals were examined by the thermal and mechanical paw tests. Different animal groups were used for the experiments and they were only tested with one concentration of chemicals (AITC, CA, capsaicin) or vehicle and were not repeatedly used. Six rats were used for each group.

### 2.4 Behavioral testing

Two behavioral models were used: thermal paw withdrawal reflex and mechanical paw withdrawal reflex (IITC, Woodland Hills, CA, USA). Prior to initiating the tests, baseline values were assessed for the experimental and control rats for thermal and mechanical withdrawal tests, which involved averaging five baseline measurements for the left and right hind paws with 5 min intervals.

#### 2.4.1 Thermal paw withdrawal (Hargreaves) test

Rats were first habituated over three successive daily sessions to stand on a glass surface heated to 30±1 °C within a ventilated Plexiglas enclosure. Before formal testing, baseline latencies for paw withdrawals evoked by radiant thermal stimulation were measured five times per paw, with at least 5 min intervals between tests of a given paw. A light beam (Plantar Test 390, IITC) was focused onto the plantar surface of the hindpaw through a glass plate from below, and the latency from onset of the light to brisk withdrawal of the stimulated paw was measured. To prevent potential tissue damage, a cutoff time of 20 s was used if no paw movement occurred. Withdrawal latencies for both the treated and untreated paws were measured 5, 15, 30, 45, 60, and 120 min after applications of AITC, CA, capsaicin, or vehicle, to the same hindpaw.

#### 2.4.2 Mechanical paw withdrawal (von Frey) test

Baseline mechanical withdrawal thresholds were assessed using an electronic von Frey filament with 90 g range (1601 C, IITC) pressed against the plantar surface of one hindpaw. This device registered the force (g) at the moment that the hindpaw was withdrawn from the filament. Following application of AITC, CA, capsaicin, or vehicle, the mechanical paw withdrawal thresholds were measured at the same post application times, as noted above for thermal paw withdrawals.

### 2.5 Data analysis

Latencies of the thermal withdrawal responses and thresholds of the mechanical responses were normalized to baseline averages and analyzed by analysis of variance with Tukey–Kramer *post-hoc* tests, using InStat 3.05 (GraphPad Software Inc., USA) software. A 95% confidence interval was used for all statistical comparisons, and the error reported is the standard error of the mean (s.e.m.).
